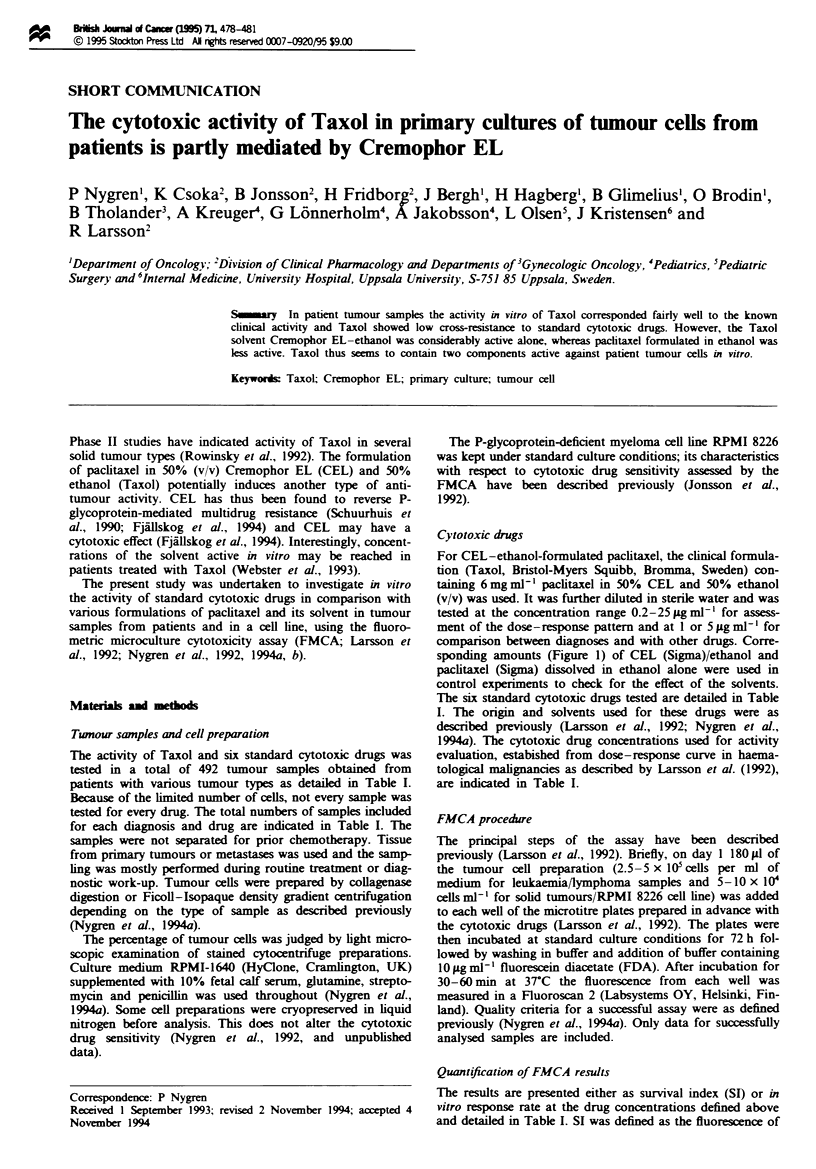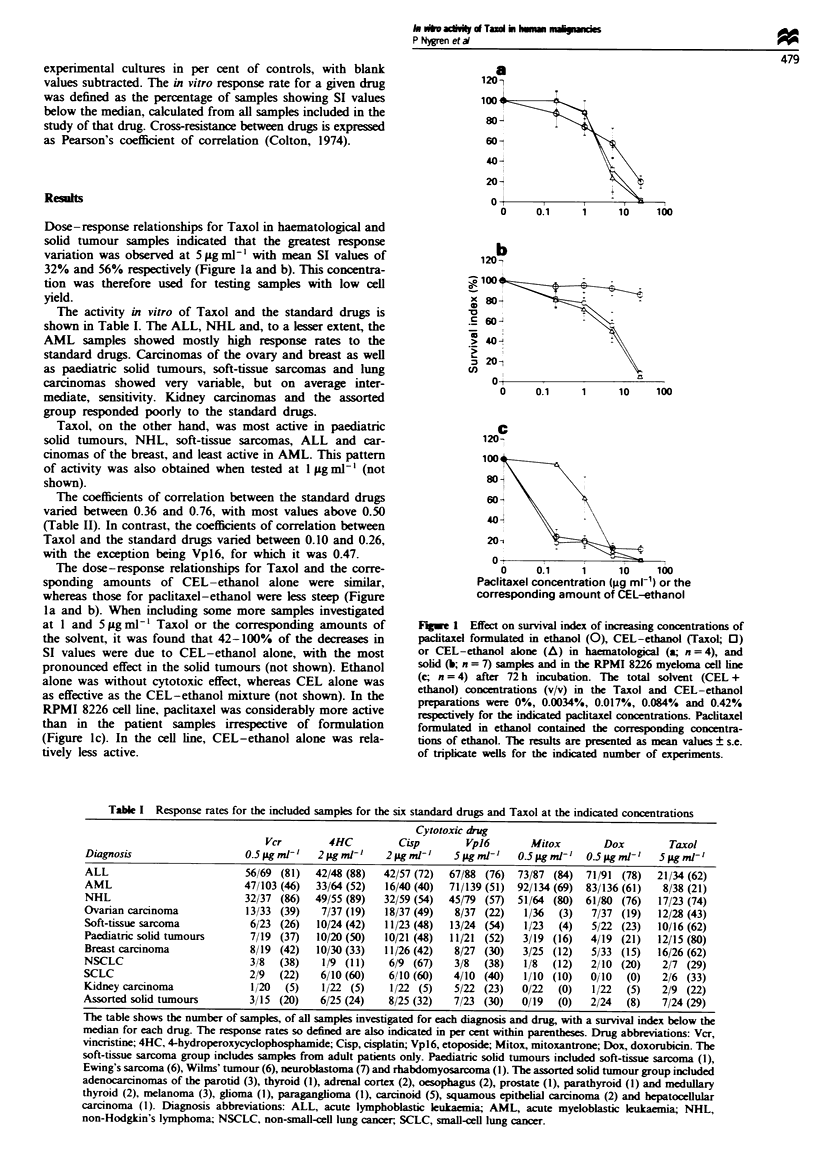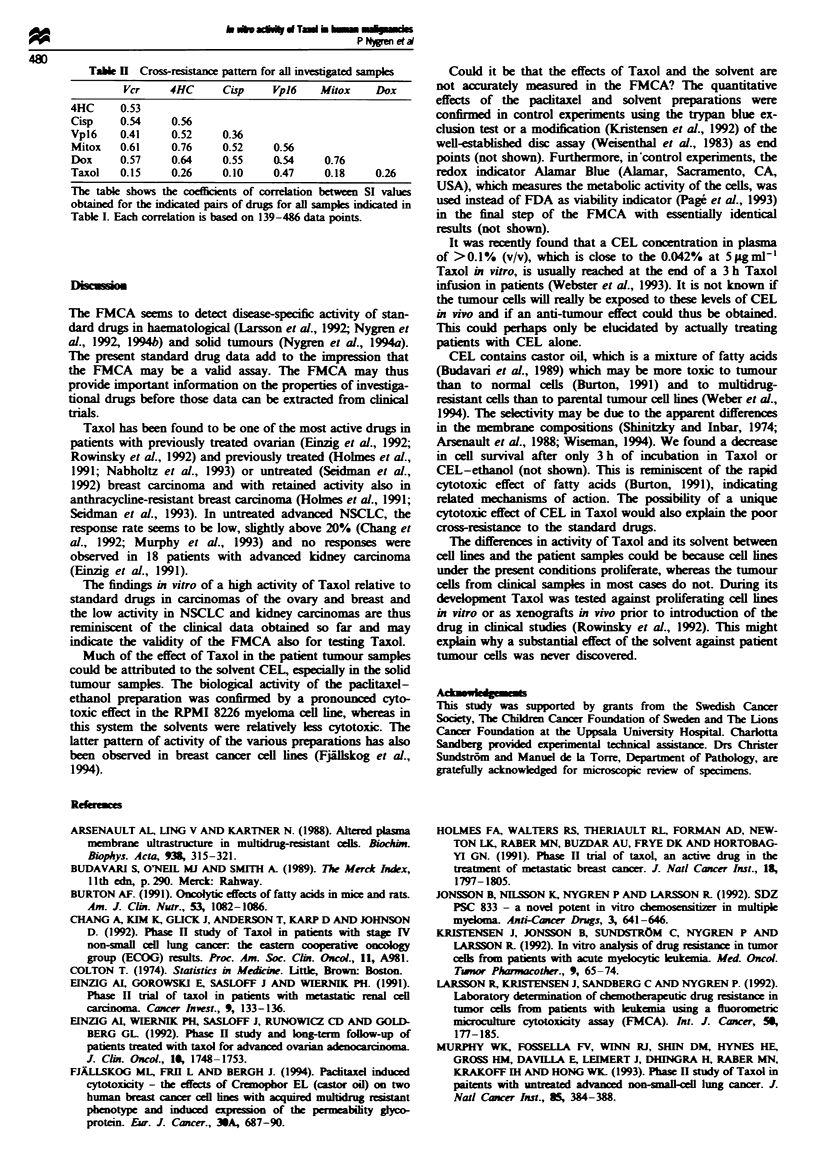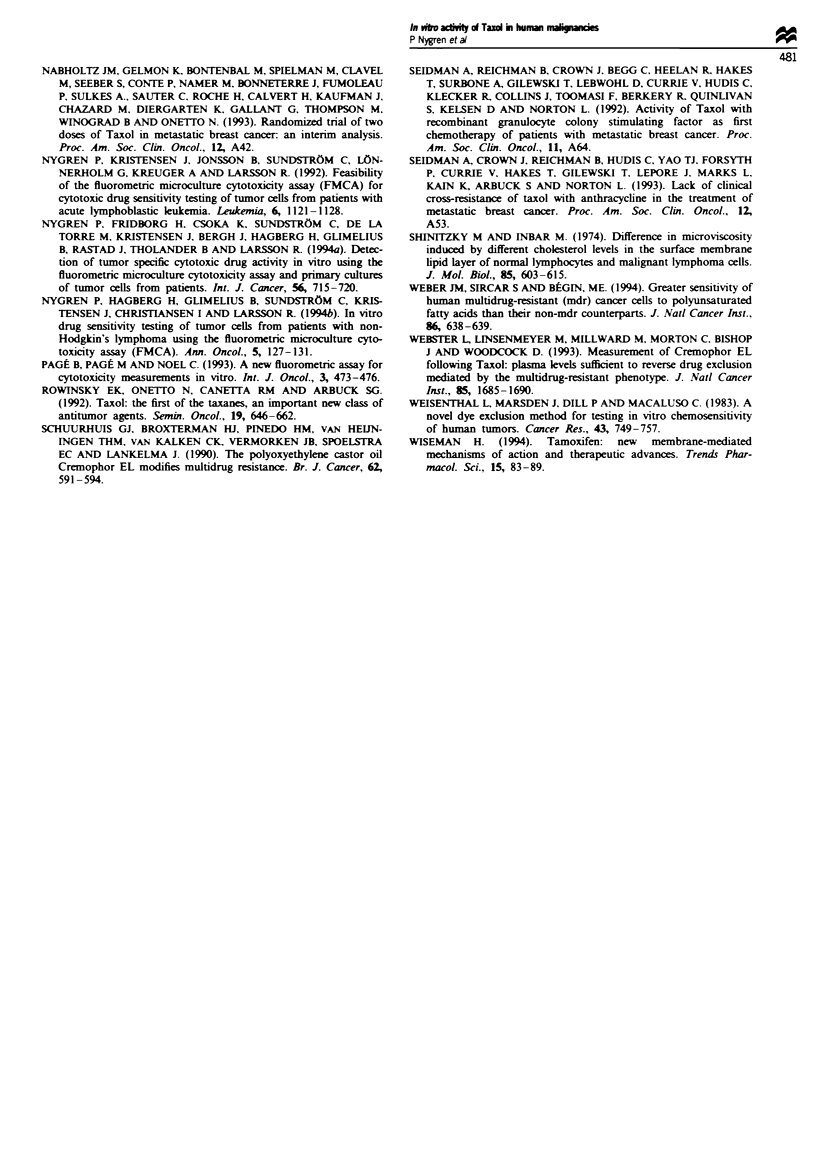# The cytotoxic activity of Taxol in primary cultures of tumour cells from patients is partly mediated by Cremophor EL.

**DOI:** 10.1038/bjc.1995.97

**Published:** 1995-03

**Authors:** P. Nygren, K. Csoka, B. Jonsson, H. Fridborg, J. Bergh, H. Hagberg, B. Glimelius, O. Brodin, B. Tholander, A. Kreuger

**Affiliations:** Department of Oncology, University Hospital, Uppsala University, Sweden.

## Abstract

In patient tumour samples the activity in vitro of Taxol corresponded fairly well to the known clinical activity and Taxol showed low cross-resistance to standard cytotoxic drugs. However, the Taxol solvent Cremophor EL--ethanol was considerably active alone, whereas paclitaxel formulated in ethanol was less active. Taxol thus seems to contain two components active against patient tumour cells in vitro.


British Journal of Cancer (1995) 71, 478–481

© 1995 Stockton Press Ltd All rights reserved 0007–0920/95 $9.00

SHORT COMMUNICATION

### The cytotoxic activity of Taxol in primary cultures of tumour cells from patients is partly mediated by Cremophor EL

P Nygren[1], K Csoka[2], B Jonsson[2], H Fridborg[2], J Bergh[1], H Hagberg[1], B Glimelius[1], O Brodin[1], B Tholander[3], A Kreuger[4], G Lönnerholm[4], Å Jakobsson[4], L Olsen[5], J Kristensen[6] and R Larsson[2]

[1]*Department of Oncology;* [2]*Division of Clinical Pharmacology and Departments of* [3]*Gynecologic Oncology,* [4]*Pediatrics,* [5]*Pediatric Surgery and* [6]*Internal Medicine, University Hospital, Uppsala University, S-751 85 Uppsala, Sweden.*

**Summary** In patient tumour samples the activity *in vitro* of Taxol corresponded fairly well to the known clinical activity and Taxol showed low cross-resistance to standard cytotoxic drugs. However, the Taxol solvent Cremophor EL–ethanol was considerably active alone, whereas paclitaxel formulated in ethanol was less active. Taxol thus seems to contain two components active against patient tumour cells *in vitro*.

**Keywords:** Taxol; Cremophor EL; primary culture; tumour cell

Phase II studies have indicated activity of Taxol in several solid tumour types (Rowinsky *et al.*, 1992). The formulation of paclitaxel in 50% (v/v) Cremophor EL (CEL) and 50% ethanol (Taxol) potentially induces another type of anti-tumour activity. CEL has thus been found to reverse P-glycoprotein-mediated multidrug resistance (Schuurhuis *et al.*, 1990; Fjällskog *et al.*, 1994) and CEL may have a cytotoxic effect (Fjällskog *et al.*, 1994). Interestingly, concentrations of the solvent active *in vitro* may be reached in patients treated with Taxol (Webster *et al.*, 1993).

The present study was undertaken to investigate *in vitro* the activity of standard cytotoxic drugs in comparison with various formulations of paclitaxel and its solvent in tumour samples from patients and in a cell line, using the fluorometric microculture cytotoxicity assay (FMCA; Larsson *et al.*, 1992; Nygren *et al.*, 1992, 1994*a*, *b*).

### Materials and methods

### *Tumour samples and cell preparation*

The activity of Taxol and six standard cytotoxic drugs was tested in a total of 492 tumour samples obtained from patients with various tumour types as detailed in Table I. Because of the limited number of cells, not every sample was tested for every drug. The total numbers of samples included for each diagnosis and drug are indicated in Table I. The samples were not separated for prior chemotherapy. Tissue from primary tumours or metastases was used and the sampling was mostly performed during routine treatment or diagnostic work-up. Tumour cells were prepared by collagenase digestion or Ficoll–Isopaque density gradient centrifugation depending on the type of sample as described previously (Nygren *et al.*, 1994*a*).

The percentage of tumour cells was judged by light microscopic examination of stained cytocentrifuge preparations. Culture medium RPMI-1640 (HyClone, Cramlington, UK) supplemented with 10% fetal calf serum, glutamine, streptomycin and penicillin was used throughout (Nygren *et al.*, 1994*a*). Some cell preparations were cryopreserved in liquid nitrogen before analysis. This does not alter the cytotoxic drug sensitivity (Nygren *et al.*, 1992, and unpublished data).

Correspondence: P Nygren
Received 1 September 1993; revised 2 November 1994; accepted 4 November 1994

The P-glycoprotein-deficient myeloma cell line RPMI 8226 was kept under standard culture conditions; its characteristics with respect to cytotoxic drug sensitivity assessed by the FMCA have been described previously (Jonsson *et al.*, 1992).

### *Cytotoxic drugs*

For CEL–ethanol-formulated paclitaxel, the clinical formulation (Taxol, Bristol-Myers Squibb, Bromma, Sweden) containing 6 mg ml$^{-1}$ paclitaxel in 50% CEL and 50% ethanol (v/v) was used. It was further diluted in sterile water and was tested at the concentration range 0.2–25 μg ml$^{-1}$ for assessment of the dose–response pattern and at 1 or 5 μg ml$^{-1}$ for comparison between diagnoses and with other drugs. Corresponding amounts (Figure 1) of CEL (Sigma)/ethanol and paclitaxel (Sigma) dissolved in ethanol alone were used in control experiments to check for the effect of the solvents. The six standard cytotoxic drugs tested are detailed in Table I. The origin and solvents used for these drugs were as described previously (Larsson *et al.*, 1992; Nygren *et al.*, 1994*a*). The cytotoxic drug concentrations used for activity evaluation, estabished from dose–response curve in haematological malignancies as described by Larsson *et al.* (1992), are indicated in Table I.

### *FMCA procedure*

The principal steps of the assay have been described previously (Larsson *et al.*, 1992). Briefly, on day 1 180 μl of the tumour cell preparation (2.5–5 × $10^5$ cells per ml of medium for leukaemia/lymphoma samples and 5–10 × $10^4$ cells ml$^{-1}$ for solid tumours/RPMI 8226 cell line) was added to each well of the microtitre plates prepared in advance with the cytotoxic drugs (Larsson *et al.*, 1992). The plates were then incubated at standard culture conditions for 72 h followed by washing in buffer and addition of buffer containing 10 μg ml$^{-1}$ fluorescein diacetate (FDA). After incubation for 30–60 min at 37°C the fluorescence from each well was measured in a Fluoroscan 2 (Labsystems OY, Helsinki, Finland). Quality criteria for a successful assay were as defined previously (Nygren *et al.*, 1994*a*). Only data for successfully analysed samples are included.

### *Quantification of FMCA results*

The results are presented either as survival index (SI) or *in vitro* response rate at the drug concentrations defined above and detailed in Table I. SI was defined as the fluorescence of experimental cultures in per cent of controls, with blank values subtracted. The *in vitro* response rate for a given drug was defined as the percentage of samples showing SI values below the median, calculated from all samples included in the study of that drug. Cross-resistance between drugs is expressed as Pearson's coefficient of correlation (Colton, 1974).

### Results

Dose-response relationships for Taxol in haematological and solid tumour samples indicated that the greatest response variation was observed at 5 $\mu g\ ml^{-1}$ with mean SI values of 32% and 56% respectively (Figure 1a and b). This concentration was therefore used for testing samples with low cell yield.

The activity *in vitro* of Taxol and the standard drugs is shown in Table I. The ALL, NHL and, to a lesser extent, the AML samples showed mostly high response rates to the standard drugs. Carcinomas of the ovary and breast as well as paediatric solid tumours, soft-tissue sarcomas and lung carcinomas showed very variable, but on average intermediate, sensitivity. Kidney carcinomas and the assorted group responded poorly to the standard drugs.

Taxol, on the other hand, was most active in paediatric solid tumours, NHL, soft-tissue sarcomas, ALL and carcinomas of the breast, and least active in AML. This pattern of activity was also obtained when tested at 1 $\mu g\ ml^{-1}$ (not shown).

The coefficients of correlation between the standard drugs varied between 0.36 and 0.76, with most values above 0.50 (Table II). In contrast, the coefficients of correlation between Taxol and the standard drugs varied between 0.10 and 0.26, with the exception being Vp16, for which it was 0.47.

The dose-response relationships for Taxol and the corresponding amounts of CEL-ethanol alone were similar, whereas those for paclitaxel-ethanol were less steep (Figure 1a and b). When including some more samples investigated at 1 and 5 $\mu g\ ml^{-1}$ Taxol or the corresponding amounts of the solvent, it was found that 42-100% of the decreases in SI values were due to CEL-ethanol alone, with the most pronounced effect in the solid tumours (not shown). Ethanol alone was without cytotoxic effect, whereas CEL alone was as effective as the CEL-ethanol mixture (not shown). In the RPMI 8226 cell line, paclitaxel was considerably more active than in the patient samples irrespective of formulation (Figure 1c). In the cell line, CEL-ethanol alone was relatively less active.

**Figure 1** Effect on survival index of increasing concentrations of paclitaxel formulated in ethanol (○), CEL-ethanol (Taxol; □) or CEL-ethanol alone (△) in haematological (**a**; $n = 4$), and solid (**b**; $n = 7$) samples and in the RPMI 8226 myeloma cell line (**c**; $n = 4$) after 72 h incubation. The total solvent (CEL + ethanol) concentrations (v/v) in the Taxol and CEL-ethanol preparations were 0%, 0.0034%, 0.017%, 0.084% and 0.42% respectively for the indicated paclitaxel concentrations. Paclitaxel formulated in ethanol contained the corresponding concentrations of ethanol. The results are presented as mean values ± s.e. of triplicate wells for the indicated number of experiments.

**Table I** Response rates for the included samples for the six standard drugs and Taxol at the indicated concentrations

| | *Cytotoxic drug* | | | | | | |
|---|---|---|---|---|---|---|---|
| *Diagnosis* | *Vcr 0.5 µg ml⁻¹* | *4HC 2 µg ml⁻¹* | *Cisp 2 µg ml⁻¹* | *Vp16 5 µg ml⁻¹* | *Mitox 0.5 µg ml⁻¹* | *Dox 0.5 µg ml⁻¹* | *Taxol 5 µg ml⁻¹* |
| ALL | 56/69 (81) | 42/48 (88) | 42/57 (72) | 67/88 (76) | 73/87 (84) | 71/91 (78) | 21/34 (62) |
| AML | 47/103 (46) | 33/64 (52) | 16/40 (40) | 71/139 (51) | 92/134 (69) | 83/136 (61) | 8/38 (21) |
| NHL | 32/37 (86) | 49/55 (89) | 32/59 (54) | 45/79 (57) | 51/64 (80) | 61/80 (76) | 17/23 (74) |
| Ovarian carcinoma | 13/33 (39) | 7/37 (19) | 18/37 (49) | 8/37 (22) | 1/36 (3) | 7/37 (19) | 12/28 (43) |
| Soft-tissue sarcoma | 6/23 (26) | 10/24 (42) | 11/23 (48) | 13/24 (54) | 1/23 (4) | 5/22 (23) | 10/16 (62) |
| Paediatric solid tumours | 7/19 (37) | 10/20 (50) | 10/21 (48) | 11/21 (52) | 3/19 (16) | 4/19 (21) | 12/15 (80) |
| Breast carcinoma | 8/19 (42) | 10/30 (33) | 11/26 (42) | 8/27 (30) | 3/25 (12) | 5/33 (15) | 16/26 (62) |
| NSCLC | 3/8 (38) | 1/9 (11) | 6/9 (67) | 3/8 (38) | 1/8 (12) | 2/10 (20) | 2/7 (29) |
| SCLC | 2/9 (22) | 6/10 (60) | 6/10 (60) | 4/10 (40) | 1/10 (10) | 0/10 (0) | 2/6 (33) |
| Kidney carcinoma | 1/20 (5) | 1/22 (5) | 1/22 (5) | 5/22 (23) | 0/22 (0) | 1/22 (5) | 2/9 (22) |
| Assorted solid tumours | 3/15 (20) | 6/25 (24) | 8/25 (32) | 7/23 (30) | 0/19 (0) | 2/24 (8) | 7/24 (29) |

The table shows the number of samples, of all samples investigated for each diagnosis and drug, with a survival index below the median for each drug. The response rates so defined are also indicated in per cent within parentheses. Drug abbreviations: Vcr, vincristine; 4HC, 4-hydroperoxycyclophosphamide; Cisp, cisplatin; Vp16, etoposide; Mitox, mitoxantrone; Dox, doxorubicin. The soft-tissue sarcoma group includes samples from adult patients only. Paediatric solid tumours included soft-tissue sarcoma (1), Ewing's sarcoma (6), Wilms' tumour (6), neuroblastoma (7) and rhabdomyosarcoma (1). The assorted solid tumour group included adenocarcinomas of the parotid (3), thyroid (1), adrenal cortex (2), oesophagus (2), prostate (1), parathyroid (1) and medullary thyroid (2), melanoma (3), glioma (1), paraganglioma (1), carcinoid (5), squamous epithelial carcinoma (2) and hepatocellular carcinoma (1). Diagnosis abbreviations: ALL, acute lymphoblastic leukaemia; AML, acute myeloblastic leukaemia; NHL, non-Hodgkin's lymphoma; NSCLC, non-small-cell lung cancer; SCLC, small-cell lung cancer.

**Table II** Cross-resistance pattern for all investigated samples

| | *Vcr* | *4HC* | *Cisp* | *Vp16* | *Mitox* | *Dox* |
|---|---|---|---|---|---|---|
| 4HC | 0.53 | | | | | |
| Cisp | 0.54 | 0.56 | | | | |
| Vp16 | 0.41 | 0.52 | 0.36 | | | |
| Mitox | 0.61 | 0.76 | 0.52 | 0.56 | | |
| Dox | 0.57 | 0.64 | 0.55 | 0.54 | 0.76 | |
| Taxol | 0.15 | 0.26 | 0.10 | 0.47 | 0.18 | 0.26 |

The table shows the coefficients of correlation between SI values obtained for the indicated pairs of drugs for all samples indicated in Table I. Each correlation is based on 139–486 data points.

### Discussion

The FMCA seems to detect disease-specific activity of standard drugs in haematological (Larsson *et al.*, 1992; Nygren *et al.*, 1992, 1994*b*) and solid tumours (Nygren *et al.*, 1994*a*). The present standard drug data add to the impression that the FMCA may be a valid assay. The FMCA may thus provide important information on the properties of investigational drugs before those data can be extracted from clinical trials.

Taxol has been found to be one of the most active drugs in patients with previously treated ovarian (Einzig *et al.*, 1992; Rowinsky *et al.*, 1992) and previously treated (Holmes *et al.*, 1991; Nabholtz *et al.*, 1993) or untreated (Seidman *et al.*, 1992) breast carcinoma and with retained activity also in anthracycline-resistant breast carcinoma (Holmes *et al.*, 1991; Seidman *et al.*, 1993). In untreated advanced NSCLC, the response rate seems to be low, slightly above 20% (Chang *et al.*, 1992; Murphy *et al.*, 1993) and no responses were observed in 18 patients with advanced kidney carcinoma (Einzig *et al.*, 1991).

The findings *in vitro* of a high activity of Taxol relative to standard drugs in carcinomas of the ovary and breast and the low activity in NSCLC and kidney carcinomas are thus reminiscent of the clinical data obtained so far and may indicate the validity of the FMCA also for testing Taxol.

Much of the effect of Taxol in the patient tumour samples could be attributed to the solvent CEL, especially in the solid tumour samples. The biological activity of the paclitaxel–ethanol preparation was confirmed by a pronounced cytotoxic effect in the RPMI 8226 myeloma cell line, whereas in this system the solvents were relatively less cytotoxic. The latter pattern of activity of the various preparations has also been observed in breast cancer cell lines (Fjällskog *et al.*, 1994).

Could it be that the effects of Taxol and the solvent are not accurately measured in the FMCA? The quantitative effects of the paclitaxel and solvent preparations were confirmed in control experiments using the trypan blue exclusion test or a modification (Kristensen *et al.*, 1992) of the well-established disc assay (Weisenthal *et al.*, 1983) as end points (not shown). Furthermore, in control experiments, the redox indicator Alamar Blue (Alamar, Sacramento, CA, USA), which measures the metabolic activity of the cells, was used instead of FDA as viability indicator (Pagé *et al.*, 1993) in the final step of the FMCA with essentially identical results (not shown).

It was recently found that a CEL concentration in plasma of >0.1% (v/v), which is close to the 0.042% at 5 $\mu$g ml$^{-1}$ Taxol *in vitro*, is usually reached at the end of a 3 h Taxol infusion in patients (Webster *et al.*, 1993). It is not known if the tumour cells will really be exposed to these levels of CEL *in vivo* and if an anti-tumour effect could thus be obtained. This could perhaps only be elucidated by actually treating patients with CEL alone.

CEL contains castor oil, which is a mixture of fatty acids (Budavari *et al.*, 1989) which may be more toxic to tumour than to normal cells (Burton, 1991) and to multidrug-resistant cells than to parental tumour cell lines (Weber *et al.*, 1994). The selectivity may be due to the apparent differences in the membrane compositions (Shinitzky and Inbar, 1974; Arsenault *et al.*, 1988; Wiseman, 1994). We found a decrease in cell survival after only 3 h of incubation in Taxol or CEL–ethanol (not shown). This is reminiscent of the rapid cytotoxic effect of fatty acids (Burton, 1991), indicating related mechanisms of action. The possibility of a unique cytotoxic effect of CEL in Taxol would also explain the poor cross-resistance to the standard drugs.

The differences in activity of Taxol and its solvent between cell lines and the patient samples could be because cell lines under the present conditions proliferate, whereas the tumour cells from clinical samples in most cases do not. During its development Taxol was tested against proliferating cell lines *in vitro* or as xenografts *in vivo* prior to introduction of the drug in clinical studies (Rowinsky *et al.*, 1992). This might explain why a substantial effect of the solvent against patient tumour cells was never discovered.

**Acknowledgements**
This study was supported by grants from the Swedish Cancer Society, The Children Cancer Foundation of Sweden and The Lions Cancer Foundation at the Uppsala University Hospital. Charlotta Sandberg provided experimental technical assistance. Drs Christer Sundström and Manuel de la Torre, Department of Pathology, are gratefully acknowledged for microscopic review of specimens.

### References

ARSENAULT AL, LING V AND KARTNER N. (1988). Altered plasma membrane ultrastructure in multidrug-resistant cells. *Biochim. Biophys. Acta*, **938**, 315–321.

BUDAVARI S, O'NEIL MJ AND SMITH A. (1989). *The Merck Index*, 11th edn, p. 290. Merck: Rahway.

BURTON AF. (1991). Oncolytic effects of fatty acids in mice and rats. *Am. J. Clin. Nutr.*, **53**, 1082–1086.

CHANG A, KIM K, GLICK J, ANDERSON T, KARP D AND JOHNSON D. (1992). Phase II study of Taxol in patients with stage IV non-small cell lung cancer: the eastern cooperative oncology group (ECOG) results. *Proc. Am. Soc. Clin. Oncol.*, **11**, A981.

COLTON T. (1974). *Statistics in Medicine*. Little, Brown: Boston.

EINZIG AI, GOROWSKI E, SASLOFF J AND WIERNIK PH. (1991). Phase II trial of taxol in patients with metastatic renal cell carcinoma. *Cancer Invest.*, **9**, 133–136.

EINZIG AI, WIERNIK PH, SASLOFF J, RUNOWICZ CD AND GOLDBERG GL. (1992). Phase II study and long-term follow-up of patients treated with taxol for advanced ovarian adenocarcinoma. *J. Clin. Oncol.*, **10**, 1748–1753.

FJÄLLSKOG ML, FRII L AND BERGH J. (1994). Paclitaxel induced cytotoxicity – the effects of Cremophor EL (castor oil) on two human breast cancer cell lines with acquired multidrug resistant phenotype and induced expression of the permeability glycoprotein. *Eur. J. Cancer.*, **30A**, 687–90.

HOLMES FA, WALTERS RS, THERIAULT RL, FORMAN AD, NEWTON LK, RABER MN, BUZDAR AU, FRYE DK AND HORTOBAGYI GN. (1991). Phase II trial of taxol, an active drug in the treatment of metastatic breast cancer. *J. Natl Cancer Inst.*, **18**, 1797–1805.

JONSSON B, NILSSON K, NYGREN P AND LARSSON R. (1992). SDZ PSC 833 – a novel potent in vitro chemosensitizer in multiple myeloma. *Anti-Cancer Drugs*, **3**, 641–646.

KRISTENSEN J, JONSSON B, SUNDSTRÖM C, NYGREN P AND LARSSON R. (1992). In vitro analysis of drug resistance in tumor cells from patients with acute myelocytic leukemia. *Med. Oncol. Tumor Pharmacother.*, **9**, 65–74.

LARSSON R, KRISTENSEN J, SANDBERG C AND NYGREN P. (1992). Laboratory determination of chemotherapeutic drug resistance in tumor cells from patients with leukemia using a fluorometric microculture cytotoxicity assay (FMCA). *Int. J. Cancer*, **50**, 177–185.

MURPHY WK, FOSSELLA FV, WINN RJ, SHIN DM, HYNES HE, GROSS HM, DAVILLA E, LEIMERT J, DHINGRA H, RABER MN, KRAKOFF IH AND HONG WK. (1993). Phase II study of Taxol in paitents with untreated advanced non-small-cell lung cancer. *J. Natl Cancer Inst.*, **85**, 384–388.

NABHOLTZ JM, GELMON K, BONTENBAL M, SPIELMAN M, CLAVEL M, SEEBER S, CONTE P, NAMER M, BONNETERRE J, FUMOLEAU P, SULKES A., SAUTER C, ROCHE H, CALVERT H, KAUFMAN J, CHAZARD M, DIERGARTEN K, GALLANT G, THOMPSON M, WINOGRAD B AND ONETTO N. (1993). Randomized trial of two doses of Taxol in metastatic breast cancer: an interim analysis. *Proc. Am. Soc. Clin. Oncol.*, **12**, A42.

NYGREN P, KRISTENSEN J, JONSSON B, SUNDSTRÖM C, LÖNNERHOLM G, KREUGER A AND LARSSON R. (1992). Feasibility of the fluorometric microculture cytotoxicity assay (FMCA) for cytotoxic drug sensitivity testing of tumor cells from patients with acute lymphoblastic leukemia. *Leukemia*, **6**, 1121-1128.

NYGREN P, FRIDBORG H, CSOKA K, SUNDSTRÖM C, DE LA TORRE M, KRISTENSEN J, BERGH J, HAGBERG H, GLIMELIUS B, RASTAD J, THOLANDER B AND LARSSON R. (1994*a*). Detection of tumor specific cytotoxic drug activity in vitro using the fluorometric microculture cytotoxicity assay and primary cultures of tumor cells from patients. *Int. J. Cancer*, **56**, 715-720.

NYGREN P, HAGBERG H, GLIMELIUS B, SUNDSTRÖM C, KRISTENSEN J, CHRISTIANSEN I AND LARSSON R. (1994*b*). In vitro drug sensitivity testing of tumor cells from patients with non-Hodgkin's lymphoma using the fluorometric microculture cytotoxicity assay (FMCA). *Ann. Oncol.*, **5**, 127-131.

PAGÉ B, PAGÉ M AND NOEL C. (1993). A new fluorometric assay for cytotoxicity measurements in vitro. *Int. J. Oncol.*, **3**, 473-476.

ROWINSKY EK, ONETTO N, CANETTA RM AND ARBUCK SG. (1992). Taxol: the first of the taxanes, an important new class of antitumor agents. *Semin. Oncol.*, **19**, 646-662.

SCHUURHUIS GJ, BROXTERMAN HJ, PINEDO HM, VAN HEIJNINGEN THM, VAN KALKEN CK, VERMORKEN JB, SPOELSTRA EC AND LANKELMA J. (1990). The polyoxyethylene castor oil Cremophor EL modifies multidrug resistance. *Br. J. Cancer*, **62**, 591-594.

SEIDMAN A, REICHMAN B, CROWN J, BEGG C, HEELAN R, HAKES T, SURBONE A, GILEWSKI T, LEBWOHL D, CURRIE V, HUDIS C, KLECKER R, COLLINS J, TOOMASI F, BERKERY R, QUINLIVAN S, KELSEN D AND NORTON L. (1992). Activity of Taxol with recombinant granulocyte colony stimulating factor as first chemotherapy of patients with metastatic breast cancer. *Proc. Am. Soc. Clin. Oncol.*, **11**, A64.

SEIDMAN A, CROWN J, REICHMAN B, HUDIS C, YAO TJ, FORSYTH P, CURRIE V, HAKES T, GILEWSKI T, LEPORE J, MARKS L, KAIN K, ARBUCK S AND NORTON L. (1993). Lack of clinical cross-resistance of taxol with anthracycline in the treatment of metastatic breast cancer. *Proc. Am. Soc. Clin. Oncol.*, **12**, A53.

SHINITZKY M AND INBAR M. (1974). Difference in microviscosity induced by different cholesterol levels in the surface membrane lipid layer of normal lymphocytes and malignant lymphoma cells. *J. Mol. Biol.*, **85**, 603-615.

WEBER JM, SIRCAR S AND BÉGIN, ME. (1994). Greater sensitivity of human multidrug-resistant (mdr) cancer cells to polyunsaturated fatty acids than their non-mdr counterparts. *J. Natl Cancer Inst.*, **86**, 638-639.

WEBSTER L, LINSENMEYER M, MILLWARD M, MORTON C, BISHOP J AND WOODCOCK D. (1993). Measurement of Cremophor EL following Taxol: plasma levels sufficient to reverse drug exclusion mediated by the multidrug-resistant phenotype. *J. Natl Cancer Inst.*, **85**, 1685-1690.

WEISENTHAL L, MARSDEN J, DILL P AND MACALUSO C. (1983). A novel dye exclusion method for testing in vitro chemosensitivity of human tumors. *Cancer Res.*, **43**, 749-757.

WISEMAN H. (1994). Tamoxifen: new membrane-mediated mechanisms of action and therapeutic advances. *Trends Pharmacol. Sci.*, **15**, 83-89.